# Reduced nest development of reared *Bombus terrestris* within apiary dense human-modified landscapes

**DOI:** 10.1038/s41598-021-82540-6

**Published:** 2021-02-12

**Authors:** Ivan Meeus, Laurian Parmentier, Matti Pisman, Dirk C. de Graaf, Guy Smagghe

**Affiliations:** 1 Department of Plants and Crops, Faculty of Bioscience Engineering, Ghent University, Coupure Links 653, 9000 Ghent, Belgium; 2 Laboratory of Molecular Entomology and Bee Pathology, Faculty of Sciences, Ghent University, Krijgslaan 281, S2, 9000 Ghent, Belgium

**Keywords:** Environmental sciences, Ecosystem ecology, Ecosystem services

## Abstract

Wild bees are in decline on a local to global scale. The presence of managed honey bees can lead to competition for resources with wild bee species, which has not been investigated so far for human-modified landscapes. In this study we assess if managed honey bee hive density influence nest development (biomass) of bumble bees, an important trait affecting fitness. We hypothesize that domesticated honey bees can negatively affect *Bombus terrestris* nest development in human-modified landscapes. In Flanders, Belgium, where such landscapes are dominantly present, we selected 11 locations with landscape metrics ranging from urban to agricultural. The bee hive locations were mapped and each location contained one apiary dense (AD) and one apiary sparse (AS) study site (mean density of 7.6 ± 5.7 managed honey bee hives per km^2^ in AD sites). We assessed the effect of apiary density on the reproduction of reared *B. terrestris* nests. Reared *B. terrestris* nests had more biomass increase over 8 weeks in apiary sparse (AS) sites compared to nests located in apiary dense (AD) sites. This effect was mainly visible in urban locations, where nest in AS sites have 99.25 ± 60.99 g more biomass increase compared to nest in urban AD sites. Additionally, we found that managed bumble bee nests had higher biomass increase in urban locations. We conclude that the density of bee hives is a factor to consider in regard to interspecific competition between domesticated honey bees and bumble bees.

## Introduction

Pollination is a key ecosystem function, as 87.5% of all wild plant species^1^ and 75% of the leading global food crops rely on animal pollination, accounting for 35% of the total global production volumes^2^. Despite their importance, wild insect pollinators are declining on a local to global scale^3–6^. The potential reduced pollination service in crops after losses of wild bees can be counteracted through integrated crop pollination (ICP), which supplements wild bee pollination services using managed pollinators. The managed honey bee (*Apis mellifera*) is an essential component herein^7^. However, at the same time managed honey bees can compete with wild bees due to niche overlap^8,9^, potentially collecting or even depleting floral resources that would otherwise be available for wild bees^10,11^.

In order to conserve wild pollinator biodiversity and its associated pollination services^12^ it is important to determine if competition between managed honey bees and wild bees is present. Thomson^13,14^ described a correlation between an increase of feral *A. mellifera* and a decline of wild bumble bees in natural habitats; yet only after incorporation of the abiotic factor drought, as it severely influenced temporal flower composition. While in lowland pasture/arable land of Scotland the worker mean thorax widths of four bumble bee species (thorax widths is here an indicator of resource availability during the larval stage) was lower in sample sites where honey bees were present compared to sites were they were absent^15^.

Recent studies on the interaction of wild bees and honey bees focused on natural environments with honey bees as feral bees^16^ and on environments with insect pollinated crops where managed honey bees were used as pollinators^17,18^. In the latter, managed honey bee hives are introduced in the crop field, such as rapeseed, and the managed honey bees interact with wild pollinators both within the field^19^ and sometimes in semi-natural elements around the crop field where they can influence natural plant-wild pollinator interactions^20,21^. However, the latter situations only describe specific cases where high densities (> 2 beehives/acre) of managed honey bees are used in order to perform crop pollination services. Yet, for the most common beekeeping practices, i.e. honey production, beekeepers will try to have a quality location where the hive can develop well, and thus avoid placing large densities of hives in one location.

Intra- and interspecific competition between bees depends on the limiting resource and the competition coefficient, where competition becomes apparent as the population sizes of the competing species approaches the carrying capacity of the habitat^22^. It can be expected that under common beekeeping practices, managed honey bee hive density is diluted over a larger area, resulting in reduced competitive interactions. However, no studies so far have addressed the potential competition between managed honey bees and wild bees under these densities in human-modified landscapes where honeybees are native bee species. Yet, determining the impact of managed honey bee competition in human-modified environments is important as these are increasing with the expansion of the human population worldwide^23^.

In this study, we want to assess whether moderate managed honey bee hive density has a negative impact on bumble bee colonies in human-modified landscapes such as Flanders, Belgium. We opted for a coupled study design of paired study sites within a same landscape, but with different densities of managed honey bee hives. We hypothesize that in the areas with fewer managed honey bees a better colony build-up of wild bees will be seen. Hereto, we followed nest development of artificially placed reared *Bombus terrestris* colonies in the paired apiary dense (AD) and apiary sparse (AS) study sites. The carrying capacity of the landscape to support managed honey bees and sympatric wild bees is a factor to consider. This is determined by spatial variation of landscapes and flower availability on a local and broader scale^24,25^. This aspect is included by selecting a range of urban to rural landscapes. We included both landscape types as their impact for bees can differ due to the relative importance of floral and nesting resources available^26^, with urban landscapes being supportive for *B. terrestris* nest development^27^.

## Material and methods

### Coupled design of locations with apiary dense (AD) and apiary sparse (AS) sites

Study locations are located in Flanders (Belgium) and each location contains one apiary dense (AD) and one apiary sparse (AS) site, with a distance of 1.5 ± 0.1 km between the centres of the AS and AD study sites. This distance had two rationale. Honey bee foraging distance, depending on landscape complexity and flower patch rewards, can exceed 1.5 kms^28^. Yet the probability of flower visitation decreases non-linearly with distance from the hive, with a large majority of the foraging trips within a radius of 750 m, based on waggle dance decoding of honey bees in such landscapes (average distances 633–740 m)^29–31^. Thus, we aimed to achieve a large distance between sites to reduce the probability of spillover from the apiary dense sites to the apiary sparse sites. In contrast, we aimed to keep the distance small enough to assure equal landscape metrics between paired sites. We acknowledge that some of the managed honey bees from the AD sites will forage at the AS sites, but that their total abundance will be lower (as shown in Table 1).

**Table 1:** Overview of all the locations and the number of managed honey bee hives and honey bees specimens counted in apiary dense (AD) and apiary sparse (AS) study sites.

Year	Location	No. managed honey bee hives		Honey bee count			
		AD	AS	AD		AS	
2013	G1	*4*	*0*	*nd*		*nd*	
	G2	*21*	*0*	*31*		*0*	
	Roe	*19*	*0*	*52*		*0*	
	Zing	*3*	*0*	*11*		*1*	
	Waar	*7*	*0*	*15*		*1*	
	Hore	*8*	*0*	*28*		*8*	
2015	G2	*12*	*0*	*61*	52	*8*	18
	HB	*4*	*0*	*7*	71	*3*	31
	PM	*40*	*3*	*132*	130	*20*	22
	MG	*7*	*0*	*11*	59	*2*	27
	SB	*8*	*0*	*18*	21	*6*	6
	W	*16*	*0*	*16*	103	*6*	23

nd = not determined (pan trapping failed at this location); honey bee counts are performed by pan trapping (sum of three pan trap sets over eight weeks per study site); underlined number are counts by transect walks (sum of three 50 m transects per study site).

### Counting of managed honey bee hives and honey bee specimens

Hive counts were based on registrations of apiaries at the Federal Agency for the Safety of the Food Chain. This list was used to generate a map of study sites presumed to contain many apiaries. In an area with a radius of three kilometres we contacted all registered beekeepers to obtain information on the amount of managed honey bee hives around central hives chosen in all study sites, and walked around screening for any potential unregistered apiaries in their neighbourhood. Based on this map, we selected six locations in 2013 and six locations in 2015 which met our criteria. The number of managed honey bee hives within a 750 m radius of the selected AD study sites ranged from 3 to 40 (or 1.7 to 22.6 hives/km^2^), and was always higher in the AD sites compared to the paired AS site (see Table 1). These managed honey bee hive densities are comparable in terms of European hive density averages^32^. Over all experiments, the AD sites have a mean density of 7.6 ± 5.7 bee hives per km^2^, while in Belgium the mean number of managed honey bee hives per km^2^ is 3.6^32^.

Honey bee specimen counts are based on pan trapping (2013 and 2015) and transect walks (2015). Within each study site we placed three sets of pan traps (colored funnels) at a distance of 100 ± 50 m from the centre of the study site. Each set contained three pan traps, with the following three colours: white, yellow and blue^33^. The distance between the pan traps within one set ranged from 3 to 5 m based on earlier experience and literature^34^. Each set was placed at a certain height, ranging from 0 to 0.8 m, depending on the dominant vegetation height present.

The pan traps (of 20 cm diameter) were filled with 400 ml of water, a drop of detergent with 10% formaldehyde solution to avoid putrefaction. The total survey time was 8 weeks (half of May 2015 until half July 2015) and pan traps were checked and emptied at intervals of 5 ± 2 days. The pan traps were refilled if needed. The collected specimens were temporarily frozen in − 20 °C fridge and honey bees were identified^35^. We recorded the sum of all counted honey bees per site.

In 2015 honey bee abundance was estimated by transect walks. Bees were monitored in three 50 m transects per study site. A random location was chosen within a radius of 100 ± 50 m from the centre of the study site, during dry, warm (> 15 °C) and sunny conditions between 9:30 and 18.00 h. Each transect was visited for one hour, separated over three time periods in May, June and July. Thus, we monitored 9 h over 3 transects per study site (total 108 h for all locations). Transects encompassed gardens and road verges chosen to represent similar spatial heterogeneity and comparable landscape elements within one location. Vegetation was recorded to assure that local vegetation community was similar between coupled sites (as shown in supporting information S1, giving details on the flowers presence at each transect). Paired sites were sampled on the same day. We used the total count over the 3 × 50 m walks as a measure for honey bee abundance.

### Landscape metrics of the sites within the different locations

A landscape analysis was performed within a radius of 750 m of all bumble bee hives. Land cover data was retrieved from the Biological Valuation Map (BVM) of Flanders^36^ and analysed in QGIS^37^. BVM categories were grouped in six land cover categories, based on the resources they provide for bees. The following land cover categories were identified: (1) “Semi-natural positive”, encompassing all semi-natural habitats (grasslands, woodlands, linear elements…) which can provide food or nesting substrate for wild bees, (2) “Semi-natural neutral”, all semi-natural elements that do not provide food or nesting (e.g. ponds, temporary species-poor grasslands…), (3) “Urban”, defined as the percentage of build-up, industrial areas and roads, (4) “Acres”, encompassing all agricultural areas which produce crops that do not rely on pollinators (grain, potatoes, maize, beets,…), (5) Low stem orchards, describing all plantations of low stem fruit trees (e.g. apples, pear, sweet cherry,…), characterized by an intensive management and (6) high stem orchards, describing all fields containing high stem fruit trees, characterized by an extensive management.

We selected locations with a variation of different land covers, while the variation within a location (the sites) is kept minimal. This to assure that the ability of sites, to support wild bumble bees, within a location is equal. To verify this we transformed the land cover data into a similarity matrix based upon Euclidean-distance and a Permutational multivariate analysis of variance (PERMANOVA) was performed with the adonis function within the R package vegan^38^. We tested if the land cover matrix was different in relation to the factor apiary (two levels, AS and AD) and the factor landscape type (three levels, agriculture, semi urban and urban).

Reduced levels of urbanisation were mainly coupled with an increase of agricultural land cover (Supporting information S2). In 2013 we selected six locations: three urban locations (urbanisation range: 86–99%) and three agricultural locations dominated by agricultural practises (urbanisation range: 13–26%). In 2015 we selected six locations with a gradient of urbanisation present (urbanisation range: 16–90%), one location was the same as selected in 2013.

### *Bombus terrestris* nest development

Standardized bumble bee nests (*B.* *terrestris*) (n = 72) were obtained from a commercial rearing programme (Biobest, Westerlo, Belgium). Each nest contained one queen and an average of 45 workers (± 19.9 SD). As presence of parasites and viruses could potentially influence nest development, we screened a subset of 24 nests (in 2013) before they were placed in the field. We screened for *Crithidia* spp. and *Apicistis bombi*^39^, *Nosema* spp.^40^; and viruses (deformed wing virus (DWV), sacbrood virus (SBV), black queen cell virus (BQCV) and viruses of the acute-Kashmir-Israeli-complex (AKI) by MLPA^41^. DNA and RNA extract were performed on pools of 10 bumble bees per nest. No pathogens were detected; this does not ensure complete freedom from disease, but excludes that pathogens are a major contributing factor.

Nests were put into a polystyrene box for protection against cold and wet weather conditions and placed with their entrance to the east^42^. In 2013 we placed three bumble bee nests in the centre of each study site (in total 36 nests). In 2015 we placed three bumble bee nests per study site at a radius of 100 ± 50 m from the centre. In standard conditions these nests contain sugar containers, these were removed before the nests were placed outside, also access pollen (added for transport to the lab) was removed.

The development of the nests was followed over a period of six weeks (before new offspring emerge, i.e. drones and daughter queens)^43^. Biomass increase (mass difference after six week of the plastic cage, containing the brood, workers, foundress queen and nest debris. The sugar container is not included) was chosen as a measure of bumble bee nest development^42^. As well-developed nest are able to produce more new queens (gynes) at the end of the season^44^, nest size can be seen as a proxy for reproductive output and thus fitness.

### Statistical analysis of nest development

The response variable, ‘biomass increase’ was treated as a normal error distribution after a square root (x + 75) transformation. The + 75 was used to ensure that all negative values became positive. We used a linear mixed-effects model (R package lme4 version 1.1-10^45^) with three fixed factors, being apiary density (levels = 2, AS and AD), landscape type (levels = 3, urban (urbanisation 86–99%), semi-urban (39–76%) and agricultural (13–26%), see supporting information S2 for landscape categorisation) and year (levels = 2, 2013 and 2015)). We also used a second linear mixed model, where we substituted the categorical value “landscape type” with the percentage of urbanisation as covariate, to validate whether our defined sub-categories of landscape type had effect on the model outcome. In both models the random factor site (levels = 22) was included, nested within location (Levels = 11; six in 2013 and six in 2015, of which one location is the same). The effect of urbanisation and landscape type was integrated into the model to evaluate possible landscape interaction effects, yet was not an initial study objective on itself. We included the interactions apiary*year and apiary*type (categorical or continues). Dropping the interactions systematically gave slightly improved Akaike Information Criterion (AIC) and were omitted from the final model.

We used a one Sample t-test to determine if the difference in mean biomass increase per location (i.e. sum of biomass increase in AS sites minus sum of biomass increase in AD sites) is different from zero.

We calculates the effect size Cohen's *d* to visualize mean increase of biomass is AS sites compared to AD sites^46^. Here the difference between two means is divided by a pooled standard deviation ${(S}_{p})$ or (mean_AS_ − mean_AD_)/Sp.

$${S}_{p}=\sqrt{{\left({n}_{AS}-1\right){s}_{AS}^{2}+ ({n}_{AD}-1){s}_{AD}^{2}}/{({n}_{AS}+ {n}_{AD}-2)}}$$

The Confidence interval (CI) is formulated as:

$$\mathrm{CI}\left(\mathrm{d}\right)=\sqrt[]{{{{(n}_{AS}+ {n}_{AD})}/{{(n}_{AS}{n}_{AD})}+ {{d}^{2}}/{2{(n}_{AS}+ {n}_{AD})}}}$$

## Results

### Apiary density and land cover analysis

Sites in apiary-dense areas had more honey bees than sites in apiary-scarce areas (Table 1). The differences in managed honey bee hive density indeed resulted in more honey bees in AD sites compared to AS sites.

**Figure 1:**
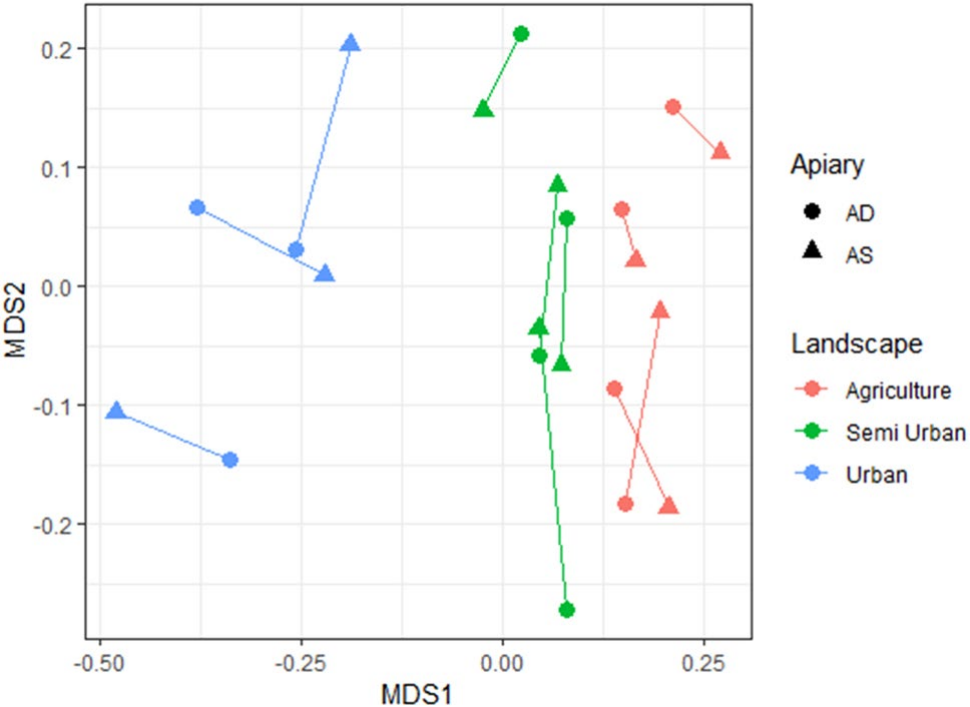
Multidimensional scaling was used to visualize the land cover dissimilarity matrix (calculation based on Euclidean distance). Circles represent AD (apiary dense) site and overlap with AS (apiary sparse) sites (rectangles) and paired sites are connected (showing that paired sites have similar land covers). The landscape types are: urban = blue, agricultural = red, and semi-urban = green.

In Fig. 1 we show that our locations have a different landscape composition, based on a matrix of six land covers. The urbanisation % was used to create three groups, called landscape types (these are agricultural, semi urban and urban locations). The landscape composition was different in the three landscape types (PERMANOVA; F _landscape type_ = 57.8; total df = 21; *P* = 0.01), while no difference was recorded within locations (factor AS versus AD) (PERMANOVA; F _apiary_ = 0.074; total df = 21; *P* = 0.87). We can conclude that the landscape composition is randomized within locations, and is not correlated with the factor apiary (AS versus AD).

### Bumble bee nest development: the factor apiary in different landscapes

**Figure 2:**
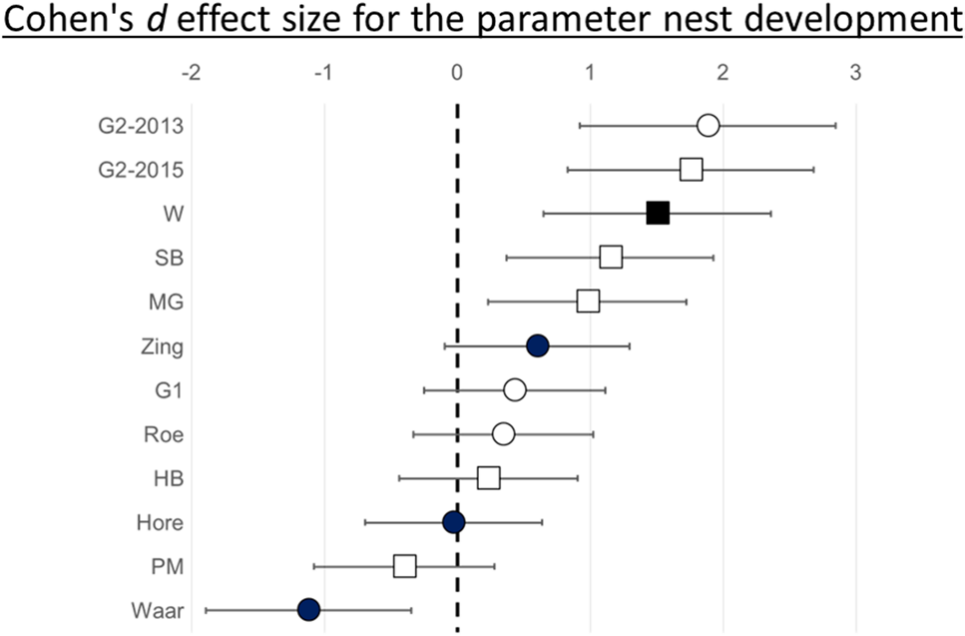
Cohen's *d* effect size are positive when the bumble bee nest biomass increase in apiary sparse (AS) sites is greater than in apiary dense (AD) sites (a proxy for higher nest fitness). Circles represent the dataset of 2013, while squares represent the dataset of 2015. The filled circles represent locations dominated with agricultural landscape elements. In each location (n = 12; 6 in 2013 and 6 in 2015) we had 2 × 3 *B. terrestris* nests (n = 72). Linear mixed-effects models are performed on individual data points (this is 3 nests per site, 2 sites (AD and AS) per location, 6 locations per year and 2 years or 3*2*6*2 = 72 data points) and not on Cohen's *d* effect sizes, which are for visualization purpose only. To calculate the effect size between AS and AD sites we used the Cohen's *d* formula^46^.

**Figure 3:**
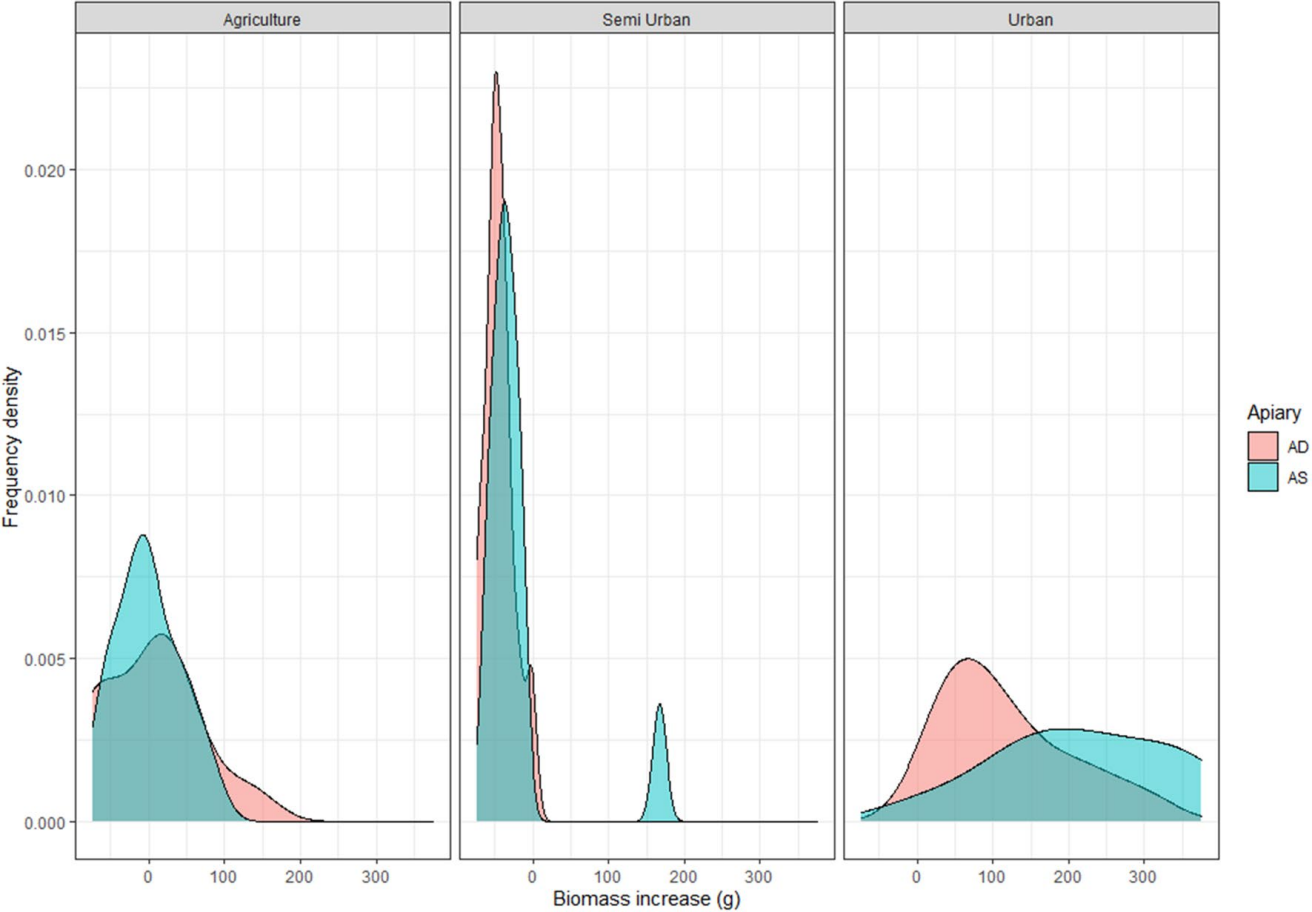
The frequency densities of the biomass increase of a bumble bee nests (in grams) in three different landscape types (Agriculture; Semi Urban, and Urban). The blue–green graphs represent apiary sparse (AS) sites, the red are apiary dense (AD) sites.

Within each location we calculated the Cohen's *d* effect size for the parameter biomass increase. This standardized difference in mean biomass increase between AS and AD site is visualized in Fig. 2). The confidence interval (CI) for five locations is above zero with effect sizes between 1.0 and 1.9. The CI in 6 locations overlaps with zero, of which 4 are positive. In one location we have an effect size of − 1.1, which is not overlapping with zero. The actual delta mean biomass increase in AS compared to AD sites is given in Table 2, which is significantly different from zero (One Sample t-test = t = 2.21, df = 11, *P* = 0.05). This higher biomass increase is mainly observed in locations where bumble bee nests developed well, like urban areas (Fig. 3).

**Table 2:** Mean biomass increase in AS sites.

Location	Year	Type	Mean delta biomass (g)* (increase in AS sites)
G1	2013	Urban	36
G2	2013	Urban	164
Waar	2013	Agricultural	− 59
Hore	2013	Agricultural	− 1
Zing	2013	Agricultural	33
Roe	2013	Urban	60
PM	2015	Semi urban	− 7
HB	2015	Semi urban	7
SB	2015	Semi urban	12
W	2015	Agricultural	14
MG	2015	Semi urban	84
G2	2015	Urban	137

*Sum of biomass increase in AS sites minus sum of biomass increase in AD sites; divided by the numbers of nests per site (3). AS = apiary sparse; AD = apiary dense.

In 2013 we had three urban locations and three agricultural locations, while in 2015 we had a gradient of urbanisation. The two fixed factors improved the model significantly (landscape type: χ^2^ = 19.31, *P* < 1 × 10^–4^; apiary χ^2^ = 4.42, *P* = 0.04). Similar results were obtained when urbanisation was included as a covariate instead of a categorical factor (urbanisation: χ^2^ = 11.0, *P* < 1 × 10^–3^; apiary χ^2^ = 4.6, *P* = 0.03). Our results support the hypothesis that bumble bee nest biomass is lower in AD sites.

### Bumble bee nest development: the factor landscape

**Figure 4:**
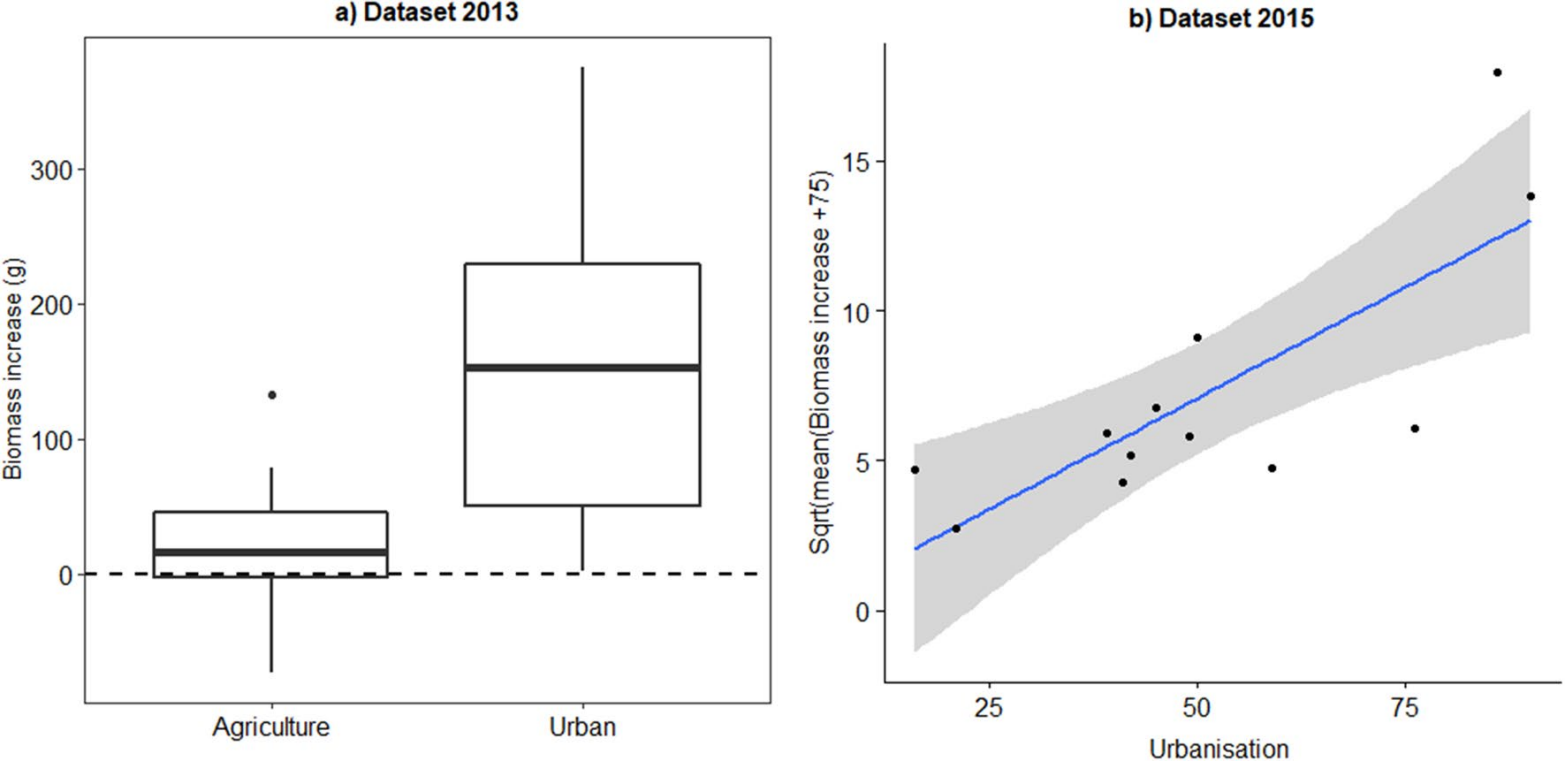
Biomass increase of bumble bee nests in relation to the land cover parameter urbanisation. (**A**) In the dataset of 2013 we had 3 urban locations while 3 locations had an agricultural character (each locations had 6 nests, with a total of 36 nests). (**B**) In 2015 the % urbanisation in the 6 locations showed a gradual increase (each locations had 6 nests, with a total of 36 nests). In both years urbanisation had a positive influence on the development of *Bombus terrestris* nests.

In 2013 we sampled two contrasting landscape types: urban locations (urbanisation between 86 and 99%) and agricultural locations (urbanisation between 13 and 26%). We found that within agricultural landscapes only a minority of nests showed a biomass increase, while in urban locations most nests had a good development (Fig. 4A). The factor landscape type (agricultural vs. urban) significantly improved the mixed model (landscape type: χ^2^ = 11.8, *P* = 0.0006). In order to have more nests with a biomass increase we selected a gradient of urbanisation in the 2015 experiment ranging from 16 to 90%. One location, with the highest urbanisation degree, was the same location as in 2013. The explanatory variable urbanisation degree was again a significant factor in the model (urbanisation χ^2^ = 8.2, *P* = 0.004). As visualized in Fig. 4B this relation is mainly driven by the four most extreme data points originating from two locations (one agricultural (< 25% urbanisation) and one urban location (> 75% urbanisation)), while the semi-urban locations (N = 4) (35% < semi-urban < 65%) have an intermediate nest development.

## Discussion

High densities of managed honey bees can potentially compete for floral resources with wild bee populations. In this study, we measured nest development of reared *B. terrestris* as a proxy for fitness in order to assess whether or not interspecific competition is present. We found that bumble bee nests placed in apiary dense sites had a reduced nest development compared to those in apiary sparse landscapes. This observation shows that colony performance suffers in areas with high densities of *A. mellifera* and suggest interspecific competition for floral resources.

Previous studies have observed fewer wild bees in areas with high densities of honey bees, yet lower wild bee counts can be a result of spatial displacement of wild bees^19^, and this must not necessarily lead to significant reductions of wild bee populations. Spatial displacement turns into competitive displacement if both species have identical niches^47^. Yet, bees have the ability to adjust their foraging preference in accordance to interspecific competition on nectar rewards^48^. We argue that these alternatives food source choices can also have a fitness cost, and indeed we observed a lower nest development of reared *B. terrestris* in apiary dense (AD) sites. At least for this specific bumble bee species niche differentiation is not prominent enough to attenuate interspecific competitive interactions with honey bees. Indeed the focal species has an overlap in flower choice^8,13^, and food resource depletion by honey bees can be expected for this species, especially in simplified landscapes^49^. But also in the urban context bumble bee flower visitation rates are negatively correlated to honey bee hive densities^50^.

### *Bombus terrestris* nests develop well within cities

In many locations (harbouring the two paired AS and AD study sites) bumble bee nests showed no net biomass increase. Also Ellis, et al.^51^ reported that, mainly in flower-poor environments, reared *B. terrestris* nests failed to develop. Within our setup, urban environments are linked with good *B. terrestris* nest development, while in agricultural sites poor or even a drop in nest biomass was observed (Fig. 2). Higher reproductive success in urban opposed to agricultural environments was previously also observed using caught wild queens to initiate nest development^27^. The impact of urbanisation on bumble bees is not a settled case; it is clear that destruction of natural habitat has a negative impact, yet a different response of functional groups toward urbanisation has been reported^52,53^. Furthermore the term urbanisation is too broad to be directly used in relation to bee development, as it can cover many different aspects of urban development^54^. A generalist pollinator, like *B. terrestris* can forage on typical garden flowers, which could lead towards a food surplus in regions with few nests and therefore good development of the reared *B. terrestris* nests. In general urbanisation is an important driver of local habitat loss, on the other hand it also provides refuges for certain bee species and functional traits^55^.

### The use of managed bumble bees compared to wild captured queens

The use of managed bumble bee queens instead of capturing wild queens to evaluate bumble bee development in relation to the landscape context has its pro and cons. The advantage is that the origin of the queens is fixed (and more standardized); thereby increasing the power of the experiment (lower variation). Plus it is more easy to initiate high numbers of nests. In contrast, it can be argued that the use of natural occurring bumble bee queens reflects more the true nature of the insect^27^. Yet in this case the capturing location of the spring queen is an additional factor which needs to be incorporated in the study design. This reduces the degrees of freedom, yet introduces a potentially interesting factor to study.

The reduced bumble bee development in apiary dense sites is mainly apparent in urban landscapes. Here the nests develop well, and work as a good measuring tool, as enough food resources were present to allow colony growth and to observe differences in growth. It can be speculated that in regions with low or no biomass increase the nests do not function as proper measuring tools to assess the effect of the landscape on its development. Meaning below a certain threshold of food availability the colony is not able to increase its workforce anymore, resulting in a negative spiral instead of a positive one. It is clear that negative growth will not result in the production of gynes (the fitness parameter for which biomass increase is the proxy).

### Future directions

A primary focus, to reduce interspecific competition between domesticated bees and bumble bees, should be on determining the carrying capacity of human-modified environments within the proximity of apiaries. Here the habitat composition in relation to managed honey bees and sympatric bumble bees needs to be considered. Although we do not present data on the mechanism behind the competitive interactions, it can be speculated that food resource competition is contributing, as pollen quality and quantity influx is a good predictor of bumble bee nest development^56^. For habitat improvements the relationship between available floral resources and bees is one of the key aspects^24^, aside from flower abundance also flower diversity will influence competitive interactions. In agricultural landscapes where the presence of bumble bees and solitary bees are limited for pollination purposes, the density of managed honey bees is factor to consider when one wants to supplement natural pollination services. In order to optimize the landscape in relation to different bee species a good surveillance of apiary placement will be important. In addition, a landscape-dependent apiary placement, which is based on floral (and nesting) resource availability, should also be beneficial to the beekeeping community, as it should lead to stronger bee hives and thus less winter losses.

## Supplementary Information

Supplementary Information 1.

Supplementary Information 2.

Supplementary Information 3.

## Data Availability

Data files are public available, supporting information 3 contains raw data of nest development.

## References

[1] Ollerton J, Winfree R, Tarrant S (2011). How many flowering plants are pollinated by animals?. Oikos.

[2] Klein AM (2007). Importance of pollinators in changing landscapes for world crops. Proc. R. Soc. B Biol. Sci..

[3] Kremen C, Williams NM, Thorp RW (2002). Crop pollination from native bees at risk from agricultural intensification. Proc. Natl. Acad. Sci. U.S.A..

[4] Potts SG (2010). Global pollinator declines: Trends, impacts and drivers. Trends Ecol. Evol..

[5] Tscharntke T (2012). Landscape moderation of biodiversity patterns and processes—Eight hypotheses. Biol. Rev..

[6] Winfree R, Aguilar R, Vazquez DP, LeBuhn G, Aizen MA (2009). A meta-analysis of bees' responses to anthropogenic disturbance. Ecology.

[7] Isaacs R (2017). Integrated crop pollination: Combining strategies to ensure stable and sustainable yields of pollination-dependent crops. Basic Appl. Ecol..

[8] Steffan-Dewenter I, Tscharntke T (2000). Resource overlap and possible competition between honey bees and wild bees in central Europe. Oecologia.

[9] Paini DR, Roberts JD (2005). Commercial honey bees (*Apis mellifera*) reduce the fecundity of an Australian native bee (*Hylaeus alcyoneus*). Biol. Cons..

[10] Schaffer WM (1983). Competition for nectar between introduced honey bees and native North American bees and ants. Ecology.

[11] Dupont YL, Hansen DM, Valido A, Olesen JM (2004). Impact of introduced honey bees on native pollination interactions of the endemic *Echium wildpretii* (Boraginaceae) on Tenerife, Canary Islands. Biol. Cons..

[12] Garibaldi LA (2013). Wild pollinators enhance fruit set of crops regardless of honey bee abundance. Science.

[13] Thomson DM (2016). Local bumble bee decline linked to recovery of honey bees, drought effects on floral resources. Ecol. Lett..

[14] Thomson D (2004). Competitive interactions between the invasive European honey bee and native bumble bees. Ecology.

[15] Goulson D, Sparrow K (2009). Evidence for competition between honeybees and bumblebees; effects on bumblebee worker size. J. Insect. Conserv..

[16] Paini DR (2004). Impact of the introduced honey bee (*Apis mellifera*) (Hymenoptera : Apidae) on native bees: A review. Austral. Ecol..

[17] Gross CL (2001). The effect of introduced honeybees on native bee visitation and fruit-set in *Dillwynia juniperina* (Fabaceae) in a fragmented ecosystem. Biol. Cons..

[18] Nielsen A, Reitan T, Rinvoll AW, Brysting AK (2017). Effects of competition and climate on a crop pollinator community. Agric. Ecosyst. Environ..

[19] Lindström SAM, Herbertssön L, Rundlof M, Bommarco R, Smith HG (2016). Experimental evidence that honeybees depress wild insect densities in a flowering crop. Proc. R. Soc. B Biol. Sci..

[20] Magrach A, González-Varo JP, Boiffier M, Vilà M, Bartomeus I (2017). Honeybee spillover reshuffles pollinator diets and affects plant reproductive success. Nat. Ecol. Evol..

[21] González-Varo JP, Vilà M (2017). Spillover of managed honeybees from mass-flowering crops into natural habitats. Biol. Conserv..

[22] Begon M, Harper JL, Townsend CR (1996). Ecology: Individuals, Populations, and Communities.

[23] United Nations. (United Nations, Department of Economic and Social Affairs, Population Division, New York, 2012).

[24] Goulson D, Nicholls E, Botias C, Rotheray EL (2015). Bee declines driven by combined stress from parasites, pesticides, and lack of flowers. Science.

[25] Scheper J (2015). Local and landscape-level floral resources explain effects of wildflower strips on wild bees across four European countries. J. Appl. Ecol..

[26] McCune F, Normandin E, Mazerolle MJ, Fournier V (2019). Response of wild bee communities to beekeeping, urbanization, and flower availability. Urban Ecosyst..

[27] Samuelson AE, Gill RJ, Brown MJF, Leadbeater E (2018). Lower bumblebee colony reproductive success in agricultural compared with urban environments. Proc. R. Soc. B Biol. Sci..

[28] Steffan-Dewenter I, Kuhn A (2003). Honeybee foraging in differentially structured landscapes. Proc. R. Soc. B Biol. Sci..

[29] Couvillon MJ, Schurch R, Ratnieks FLW (2014). Dancing bees communicate a foraging preference for rural lands in high-level agri-environment schemes. Curr. Biol..

[30] Bänsch S, Tscharntke T, Ratnieks FLW, Härtel S, Westphal C (2020). Foraging of honey bees in agricultural landscapes with changing patterns of flower resources. Agric. Ecosyst. Environ..

[31] Walther-Hellwig K, Frankl R (2000). Foraging distances of *Bombus muscorum*, *Bombus lapidarius*, and *Bombus terrestris* (Hymenoptera, Apidae). J. Insect Behav..

[32] Chauzat MP (2013). Demographics of the European apicultural industry. PLoS ONE.

[33] Stanley DA, Gunning D, Stout JC (2013). Pollinators and pollination of oilseed rape crops (*Brassica napus* L.) in Ireland: ecological and economic incentives for pollinator conservation. J. Insect Conserv..

[34] Westphal C (2008). Measuring bee diversity in different European habitats and biogeographical regions. Ecol. Monogr..

[35] Lebuhn, G., Droege, S., Connor, E., Gemmill-Herren, B. & Azzu, N. in *Guidance for practioners* 64 pp. (FAO, Rome, 2016).

[36] De Saeger, S. *et al.* (ed Rapporten van het Instituut voor Natuur- en Bosonderzoek 2016) (Instituut voor Natuur- en Bosonderzoek, Brussel, 2016).

[37] 3QGIS_Development_Team. *QGIS Geographic Information System*, 2018).

[38] Oksanen, J. *et al.* Community Ecology Package 'Vegan'. (2016). https://github.com/vegandevs/vegan.

[39] Meeus I, de Graaf DC, Jans K, Smagghe G (2010). Multiplex PCR detection of slowly-evolving trypanosomatids and neogregarines in bumblebees using broad-range primers. J. Appl. Microbiol..

[40] Ravoet J (2014). Widespread occurrence of honey bee pathogens in solitary bees. J. Invertebr. Pathol..

[41] De Smet L (2012). BeeDoctor, a versatile MLPA-based diagnostic tool for screening bee viruses. PLoS ONE.

[42] Parmentier L (2014). Commercial bumblebee hives to assess an anthropogenic environment for pollinator support: A case study in the region of Ghent (Belgium). Environ. Monit. Assess..

[43] Rundlöf M (2015). Seed coating with a neonicotinoid insecticide negatively affects wild bees. Nature.

[44] Goulson D (2003). Bumblebees: Their Behaviour and Ecology.

[45] Bates D, Machler M, Bolker BM, Walker SC (2015). Fitting linear mixed-effects models using lme4. J. Stat. Softw..

[46] Hedges L, Olkin I (1985). Statistical Methods for Meta-Analysis.

[47] DeBach P (1966). The competitive displacement and coexistence principles. Annu. Rev. Entomol..

[48] Balfour NJ, Gandy S, Ratnieks FLW (2015). Exploitative competition alters bee foraging and flower choice. Behav. Ecol. Sociobiol..

[49] Herbertssön L, Lindström SAM, Rundlof M, Bornmarco R, Smith HG (2016). Competition between managed honeybees and wild bumblebees depends on landscape context. Basic Appl. Ecol..

[50] Ropars L, Dajoz I, Fontaine C, Muratet A, Geslin B (2019). Wild pollinator activity negatively related to honey bee colony densities in urban context. PLoS ONE.

[51] Ellis C, Park KJ, Whitehorn P, David A, Goulson D (2017). The neonicotinoid insecticide Thiacloprid impacts upon bumblebee colony development under field conditions. Environ. Sci. Technol..

[52] Geslin B, Gauzens B, Thebault E, Dajoz I (2013). Plant pollinator networks along a gradient of urbanisation. PLoS ONE.

[53] Neame LA, Griswold T, Elle E (2013). Pollinator nesting guilds respond differently to urban habitat fragmentation in an oak-savannah ecosystem. Insect Conserv. Divers..

[54] Glaum P, Simao M-C, Vaidya C, Fitch G, Iulinao B (2017). Big city *Bombus*: Using natural history and land-use history to find significant environmental drivers in bumble-bee declines in urban development. R. Soc. Open Sci..

[55] Normandin E, Vereecken NJ, Buddle CM, Fournier V (2017). Taxonomic and functional trait diversity of wild bees in two urban settings. PeerJ.

[56] Moerman R, Vanderplanck M, Fournier D, Jacquemart AL, Michez D (2017). Pollen nutrients better explain bumblebee colony development than pollen diversity. Insect Conserv. Divers..

