# Supplementary material for: Reduced nest development of reared Bombus terrestris within apiary dense human-modified landscapes
Source: Sci Rep. 2021 Feb 12;11:3755. doi: 10.1038/s41598-021-82540-6 (PMC7881143; doi:10.1038/s41598-021-82540-6)
Supplement: Supplementary file 1 — Supplementary Information 1. [file 41598_2021_82540_MOESM1_ESM.docx]

Reduced nest development of reared *Bombus terrestris* within apiary dense anthropogenic landscapes

***Supplementary Material 1***

Ivan Meeus^1,*,$^ Laurian Parmentier^1,$*^, Matti Pisman^1^, Dirk C. de Graaf^2^ and Guy Smagghe^1^

^1^ Department of Plants and Crops, Faculty of Bioscience Engineering, Ghent University, Coupure Links 653, 9000 Ghent, Belgium

^2^ Laboratory of Molecular Entomology and Bee Pathology, Faculty of Sciences, Ghent University, Krijgslaan 281, S2, 9000 Ghent, Belgium

* equal first authors

$ Corresponding author:

Email: [ivan.meeus@UGent.be](mailto:ivan.meeus@UGent.be), laurian.parmentier@ugent.be

Tel: +32 9 264 6146

**Supporting information 1: flower composition of transect walks**

Transects walks were performed to count bees in coupled AD (apiary dense) and AS (apiary sparse) sites. Transects encompassed gardens and road verges. They were chosen to have a spatial heterogeneity on transect scale and are surrounded with comparable landscape elements on a location scale. Here we describe the transect vegetation, needed to assure a similar local vegetation community between paired sites within our coupled study design.

### Table S1.1. Main flowering plants blossoming along transect walks

| Location | Site | *Achilea* | *Catalpa* | *Dahlia* | *Deutzia* | *Geranium* | *Lamium* | *Lonicera* | *Rododendron* | *Rosa* | *Salvia* | *Taraxacum* | *Trifolium* |  |
| --- | --- | --- | --- | --- | --- | --- | --- | --- | --- | --- | --- | --- | --- | --- |
| PM | AD |  |  |  |  |  |  |  |  |  | x |  | x |  |
|  | AS |  |  |  |  |  |  |  |  |  | x |  | x |  |
| SB | AD |  |  |  |  |  |  |  | x | X |  |  | x |  |
|  | AS |  |  |  |  |  |  |  | x |  | x |  |  |  |
| G2 | AD |  |  |  | x | x |  | x |  |  |  | x | x |  |
|  | AS |  | x |  | x | x | x |  |  |  | x |  | x |  |
| HB | AD |  |  |  |  | x |  |  |  |  | x |  | x |  |
|  | AS |  |  | x |  | x |  |  |  |  |  |  | x |  |
| MG | AD | x |  |  | x | x |  |  | x |  |  | x | x |  |
|  | AS |  |  |  |  |  | x |  | x |  | x | x | x |  |
| W | AD |  |  |  | x |  |  |  | x | X |  |  |  |  |
|  | AS |  |  |  |  | x |  |  |  |  | x |  | x |  |

Plant species similarity between AD and AS was tested by calculating the Bray-Curtis similarity matrix and permutational multivariate analysis of variance (PERMANOVA) using 999 permutations within the call Adonis in the Vegan package of R (Anderson 2001). No difference was found between AD and AS sites (PERMANOVA; F_apiary_ = 0.3; *P* = 0.90) and locations (PERMANOVA; F_apiary_ = 1.2; *P* = 0.23); results are visualised in the 2-dimentional NMDS plots in Fig. S2.1.


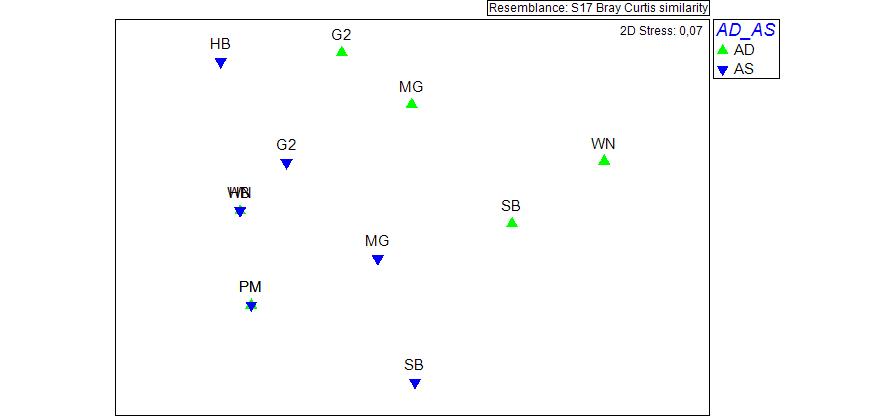


### Figure S1.1. 2-dimentional NMDS plots of observed flowering plants in AD versus AS transects

**References**

Anderson, M.J. (2001) A new method for non-parametric multivariate analysis of variance. *Austral Ecology*, **26,** 32–46.
