# Supplementary material for: Reduced nest development of reared Bombus terrestris within apiary dense human-modified landscapes
Source: Sci Rep. 2021 Feb 12;11:3755. doi: 10.1038/s41598-021-82540-6 (PMC7881143; doi:10.1038/s41598-021-82540-6)
Supplement: Supplementary file 2 — Supplementary Information 2. [file 41598_2021_82540_MOESM2_ESM.docx]

Reduced nest development of reared *Bombus terrestris* within apiary dense anthropogenic landscapes

***Supplementary Material 2***

Ivan Meeus^1,*,$^ Laurian Parmentier^1,$*^, Matti Pisman^1^, Dirk C. de Graaf^2^ and Guy Smagghe^1^

^1^ Department of Plants and Crops, Faculty of Bioscience Engineering, Ghent University, Coupure Links 653, 9000 Ghent, Belgium

^2^ Laboratory of Molecular Entomology and Bee Pathology, Faculty of Sciences, Ghent University, Krijgslaan 281, S2, 9000 Ghent, Belgium

* equal first authors

$ Corresponding author:

Email: [ivan.meeus@UGent.be](mailto:ivan.meeus@UGent.be), laurian.parmentier@ugent.be

Tel: +32 9 264 6146

**Supporting information 2: Land cover analysis of study sites**

In order to have landscapes with a different potential to support pollinator we sampled contrasting landscapes. In 2013 we had a focus on urban locations (urbanisation between 86% - 99%) and agricultural locations (urbanisation between 13% - 26%). Most variation is explained in the first component of the PCA analysis. Agricultural sites are more heterogeneous, yet all clearly separate from the urban sites; making a categorical classification into urban and agricultural justified. AD represent apiary dense sites, AS represent apiary sparse sites.


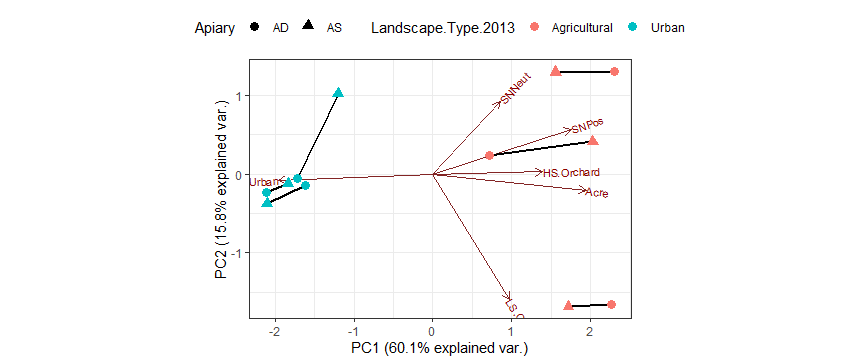


For the 2015 sampling we looked for more urban elements within agricultural sites, as all agricultural sites of 2013 showed no bumble bee nest development. This resulted in the three landscape types, agricultural, semi urban and urban. Because the latter subdivision is more arbitrary compared to the previous analysis we have chosen to also perform statistic with % urbanisation as a factor to incorporate potential landscape effects. In the main manuscript we show that urbanisation has a large effect on bumble bee nest development.


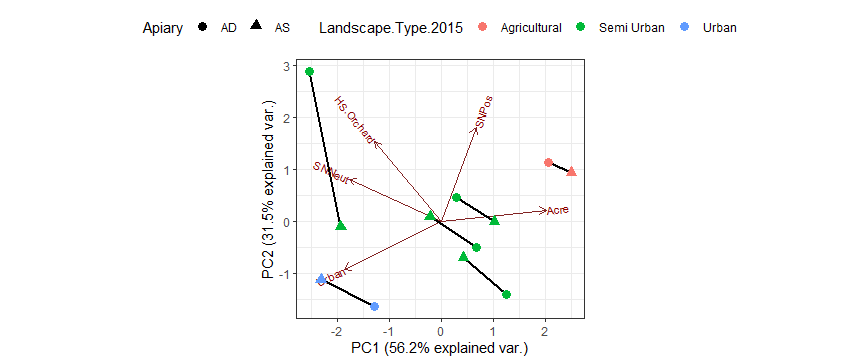


**Table S1: Percentage of land cover per study site**
