# Supplementary material for: Reduced nest development of reared Bombus terrestris within apiary dense human-modified landscapes
Source: Sci Rep. 2021 Feb 12;11:3755. doi: 10.1038/s41598-021-82540-6 (PMC7881143; doi:10.1038/s41598-021-82540-6)
Supplement: Supplementary file 3 — Supplementary Information 3. [file 41598_2021_82540_MOESM3_ESM.docx]

Reduced nest development of reared *Bombus terrestris* within apiary dense anthropogenic landscapes

***Supplementary Material 3***

Ivan Meeus^1,*,$^ Laurian Parmentier^1,$*^, Matti Pisman^1^, Dirk C. de Graaf^2^ and Guy Smagghe^1^

^1^ Department of Plants and Crops, Faculty of Bioscience Engineering, Ghent University, Coupure Links 653, 9000 Ghent, Belgium

^2^ Laboratory of Molecular Entomology and Bee Pathology, Faculty of Sciences, Ghent University, Krijgslaan 281, S2, 9000 Ghent, Belgium

* equal first authors

$ Corresponding authors:

Email: [ivan.meeus@UGent.be](mailto:ivan.meeus@UGent.be), [laurian.parmentier@ugent.be](mailto:laurian.parmentier@ugent.be)

Tel: +32 9 264 6146

**Supporting information 3:** Nest biomass increase of bumble bee nests per year, location and study site

| Location | Year | Apiary | Type | Locationnr | Site | GPS coordinates  LAT LON | | Sitenr | nest biomass increase (g) |
| --- | --- | --- | --- | --- | --- | --- | --- | --- | --- |
| G1 | 2013 | AD | Urban | 1 | Gent_ImK_(KINA)1 | 51.056 | 3.731 | 1 | 49 |
| G1 | 2013 | AD | Urban | 1 | Gent_ImK_(KINA)2 | 51.056 | 3.730 | 1 | 199 |
| G1 | 2013 | AD | Urban | 1 | Gent_ImK_(KINA)3 | 51.055 | 3.731 | 1 | 35 |
| G1 | 2013 | AS | Urban | 1 | Gent_1.5(RVT)1 | 51.042 | 3.727 | 2 | 227 |
| G1 | 2013 | AS | Urban | 1 | Gent_1.5(RVT)2 | 51.042 | 3.723 | 2 | 162 |
| G1 | 2013 | AS | Urban | 1 | Gent_1.5(RVT)3 | 51.042 | 3.728 | 2 | 2 |
| G2 | 2013 | AD | Urban | 2 | Gent_Imk_(Sterre )1 | 51.022 | 3.706 | 3 | 113 |
| G2 | 2013 | AD | Urban | 2 | Gent_Imk_(Sterre )2 | 51.063 | 3.709 | 3 | 229 |
| G2 | 2013 | AD | Urban | 2 | Gent_Imk_(Sterre )3 | 51.023 | 3.714 | 3 | 47 |
| G2 | 2013 | AS | Urban | 2 | Gent_1.5(volvo)1 | 51.033 | 3.735 | 4 | 213 |
| G2 | 2013 | AS | Urban | 2 | Gent_1.5(volvo)2 | 51.033 | 3.734 | 4 | 292 |
| G2 | 2013 | AS | Urban | 2 | Gent_1.5(volvo)3 | 51.034 | 3.736 | 4 | 375 |
| Roe | 2013 | AD | Urban | 3 | Roesel_Imk_1 | 50.942 | 3.129 | 5 | 50 |
| Roe | 2013 | AD | Urban | 3 | Roesel_Imk_2 | 50.942 | 3.128 | 5 | 52 |
| Roe | 2013 | AD | Urban | 3 | Roesel_Imk_3 | 50.942 | 3.129 | 5 | 298 |
| Roe | 2013 | AS | Urban | 3 | Roesel_1.5_1 | 50.937 | 3.140 | 6 | 350 |
| Roe | 2013 | AS | Urban | 3 | Roesel_1.5_2 | 50.938 | 3.141 | 6 | 142 |
| Roe | 2013 | AS | Urban | 3 | Roesel_1.5_3 | 50.938 | 3.138 | 6 | 89 |
| Hore | 2013 | AD | Rural | 4 | Horeb_Imk_1 | 50.833 | 3.687 | 7 | 25 |
| Hore | 2013 | AD | Rural | 4 | Horeb_Imk_2 | 50.833 | 3.688 | 7 | 21 |
| Hore | 2013 | AD | Rural | 4 | Horeb_Imk_3 | 50.832 | 3.685 | 7 | 3 |
| Hore | 2013 | AS | Rural | 4 | Horeb_1.5_1 | 50.837 | 3.703 | 8 | 5 |
| Hore | 2013 | AS | Rural | 4 | Horeb_1.5_2 | 50.836 | 3.700 | 8 | 50 |
| Hore | 2013 | AS | Rural | 4 | Horeb_1.5_3 | 50.841 | 3.706 | 8 | -8 |
| Waar | 2013 | AD | Rural | 5 | Waarsch_Imk_1 | 51.143 | 3.591 | 9 | -2 |
| Waar | 2013 | AD | Rural | 5 | Waarsch_Imk_2 | 51.144 | 3.592 | 9 | 133 |
| Waar | 2013 | AD | Rural | 5 | Waarsch_Imk_3 | 51.143 | 3.592 | 9 | 67 |
| Waar | 2013 | AS | Rural | 5 | Waarsch_1.5_1 | 51.144 | 3.568 | 10 | 43 |
| Waar | 2013 | AS | Rural | 5 | Waarsch_1.5_2 | 51.144 | 3.565 | 10 | -3 |
| Waar | 2013 | AS | Rural | 5 | Waarsch_1.5_3 | 51.145 | 3.566 | 10 | -18 |
| Zing | 2013 | AD | Rural | 6 | Zingem_Imk_1 | 50.928 | 3.590 | 11 | -73 |
| Zing | 2013 | AD | Rural | 6 | Zingem_Imk_2 | 50.927 | 3.590 | 11 | 47 |
| Zing | 2013 | AD | Rural | 6 | Zingem_Imk_3 | 50.928 | 3.589 | 11 | 12 |
| Zing | 2013 | AS | Rural | 6 | Zingem_1.5_1 | 50.929 | 3.577 | 12 | -15 |
| Zing | 2013 | AS | Rural | 6 | Zingem_1.5_2 | 50.930 | 3.578 | 12 | 21 |
| Zing | 2013 | AS | Rural | 6 | Zingem_1.5_3 | 50.929 | 3.576 | 12 | 79 |
| PM | 2015 | AD | Semi Urban | 7 | PerkMels_Imk_1 | 50.915 | 4.486 | 13 | -49 |
| PM | 2015 | AD | Semi Urban | 7 | PerkMels_Imk_2 | 50.916 | 4.487 | 13 | -48 |
| PM | 2015 | AD | Semi Urban | 7 | PerkMels_Imk_3 | 50.916 | 4.491 | 13 | -20 |
| PM | 2015 | AS | Semi Urban | 7 | PerkMels_1.5_1 | 50.924 | 4.494 | 14 | -62 |
| PM | 2015 | AS | Semi Urban | 7 | PerkMels_1.5_2 | 50.922 | 4.495 | 14 | -50 |
| PM | 2015 | AS | Semi Urban | 7 | PerkMels_1.5_3 | 50.924 | 4.494 | 14 | -26 |
| SB | 2015 | AD | Semi Urban | 8 | SteenBerg_Imk_1 | 50.914 | 4.517 | 15 | -35 |
| SB | 2015 | AD | Semi Urban | 8 | SteenBerg_Imk_2 | 50.915 | 4.520 | 15 | -51 |
| SB | 2015 | AD | Semi Urban | 8 | SteenBerg_Imk_3 | 50.913 | 4.518 | 15 | -36 |
| SB | 2015 | AS | Semi Urban | 8 | SteenBerg_1.5_1 | 50.928 | 4.535 | 16 | -41 |
| SB | 2015 | AS | Semi Urban | 8 | SteenBerg_1.5_2 | 50.930 | 4.534 | 16 | -17 |
| SB | 2015 | AS | Semi Urban | 8 | SteenBerg_1.5_3 | 50.925 | 4.535 | 16 | -27 |
| G2 | 2015 | AD | Urban | 2 | Gent_Imk_(Sterre )1 | 51.022 | 3.706 | 3 | 118 |
| G2 | 2015 | AD | Urban | 2 | Gent_Imk_(Sterre )2 | 51.063 | 3.709 | 3 | 146 |
| G2 | 2015 | AD | Urban | 2 | Gent_Imk_(Sterre )3 | 51.023 | 3.714 | 3 | 87 |
| G2 | 2015 | AS | Urban | 2 | Gent_1.5(volvo)1 | 51.033 | 3.735 | 4 | 357 |
| G2 | 2015 | AS | Urban | 2 | Gent_1.5(volvo)2 | 51.033 | 3.734 | 4 | 259 |
| G2 | 2015 | AS | Urban | 2 | Gent_1.5(volvo)3 | 51.034 | 3.736 | 4 | 145 |
| HB | 2015 | AD | Semi Urban | 9 | HuizBuiz_Imk_1 | 50.745 | 4.276 | 17 | -52 |
| HB | 2015 | AD | Semi Urban | 9 | HuizBuiz_Imk_2 | 50.746 | 4.275 | 17 | -74 |
| HB | 2015 | AD | Semi Urban | 9 | HuizBuiz_Imk_3 | 50.746 | 4.276 | 17 | -2 |
| HB | 2015 | AS | Semi Urban | 9 | HuizBuiz_1.5_1 | 50.742 | 4.252 | 18 | -57 |
| HB | 2015 | AS | Semi Urban | 9 | HuizBuiz_1.5_2 | 50.741 | 4.251 | 18 | -36 |
| HB | 2015 | AS | Semi Urban | 9 | HuizBuiz_1.5_3 | 50.741 | 4.252 | 18 | -14 |
| MG | 2015 | AD | Semi Urban | 10 | MeisGrim_Imk_1 | 50.930 | 4.345 | 19 | -67 |
| MG | 2015 | AD | Semi Urban | 10 | MeisGrim_Imk_2 | 50.931 | 4.346 | 19 | -39 |
| MG | 2015 | AD | Semi Urban | 10 | MeisGrim_Imk_3 | 50.931 | 4.343 | 19 | -59 |
| MG | 2015 | AS | Semi Urban | 10 | MeisGrim_1.5_1 | 50.933 | 4.367 | 20 | -45 |
| MG | 2015 | AS | Semi Urban | 10 | MeisGrim_1.5_2 | 50.933 | 4.369 | 20 | -36 |
| MG | 2015 | AS | Semi Urban | 10 | MeisGrim_1.5_3 | 50.934 | 4.367 | 20 | 167 |
| W | 2015 | AD | Rural | 11 | WolvNieuw_Imk_1 | 50.966 | 4.340 | 21 | -71 |
| W | 2015 | AD | Rural | 11 | WolvNieuw_Imk_2 | 50.966 | 4.341 | 21 | -71 |
| W | 2015 | AD | Rural | 11 | WolvNieuw_Imk_3 | 50.967 | 4.340 | 21 | -57 |
| W | 2015 | AS | Rural | 11 | WolvNieuw_1.5_1 | 50.978 | 4.347 | 22 | -41 |
| W | 2015 | AS | Rural | 11 | WolvNieuw_1.5_2 | 50.977 | 4.346 | 22 | -58 |
| W | 2015 | AS | Rural | 11 | WolvNieuw_1.5_3 | 50.976 | 4.347 | 22 | -58 |
